# Supplementary material for: Antimycobacterial Potential of Mycobacteriophage Under Disease-Mimicking Conditions
Source: Front Microbiol. 2020 Dec 14;11:583661. doi: 10.3389/fmicb.2020.583661 (PMC7767895; doi:10.3389/fmicb.2020.583661)
Supplement: Supplementary file 1 [file Data_Sheet_1.pdf]

Antimycobacterial potential of Mycobacteriophage under disease-mimicking conditions

Yeswanth Chakravarthy Kalapala[1], Pallavi Raj Sharma[1], Rachit Agarwal[1*]

[1]Centre for BioSystems Science and Engineering, Indian Institute of Science, Bangalore, India, 560012.

*Correspondence: rachit@iisc.ac.in

Running Title: Antimycobacterial potential of phage

Supplementary Information

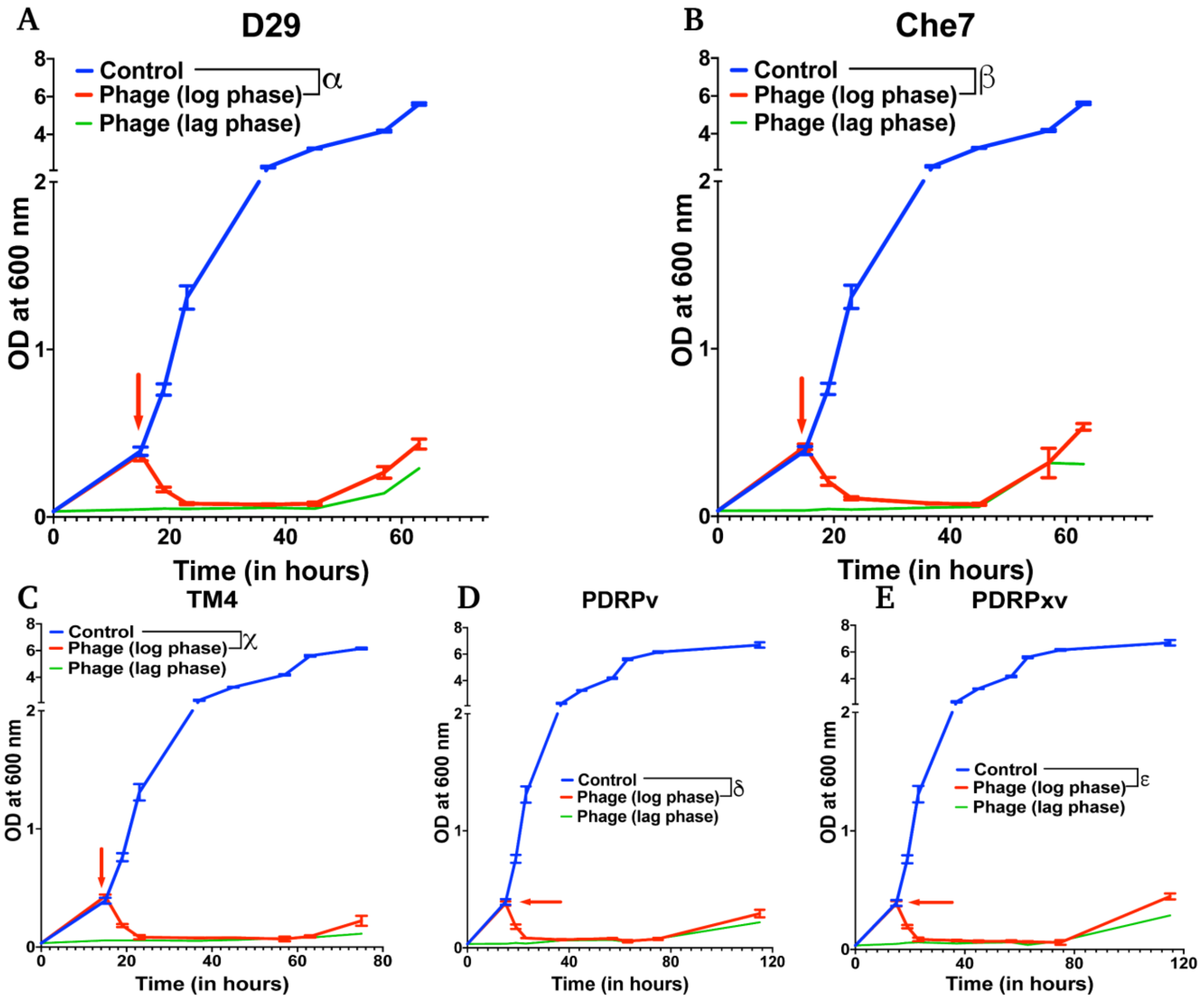

Supplementary figure S1. Growth kinetics of M *smegmatis* at MOI 30 of A) D29 B) Che7 C) TM4 D) PDRPv and E) PDRPxv**.** Red arrow represents the time point at which phage solution was added to the cultures. Multiple t-test with Holm-Sidak method was used to estimate the statistical significance. α represents a significant difference ($p<0.05$) between control and D29 treated (log phase) for all time points between 19 h and 63 h. β represents a significant difference ($p<0.05$) between control and Che7 treated (log phase) for all time points between 19 h and 63 h. χ represents a significant difference ($p<0.05$) between control and TM4 treated (log phase) for all time points between 19 h and 75 h. δ represents a significant difference ($p<0.05$) between control and PDRPv treated (log phase) for all time points between 19 h and 115 h. ε represents a significant difference ($p<0.05$) between control and PDRPxv treated (log phase) for all time points between 19 h and 115 h.

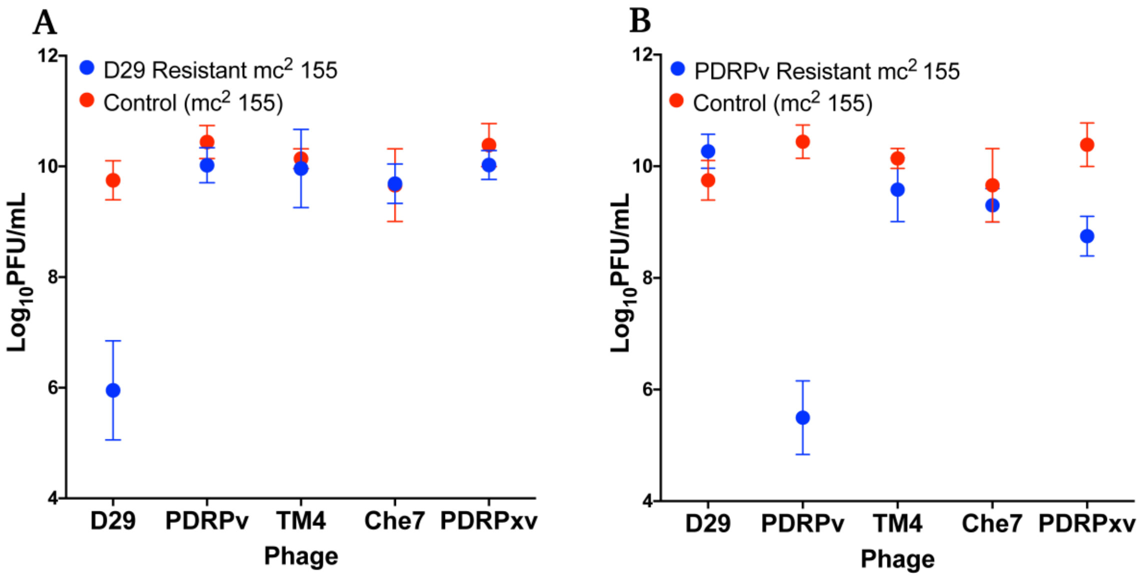

Supplementary figure S2. Phage exposure causes reduction in respective phage titer on regrowing bacteria. Phage titers of all stock phages on **A)** D29 exposed *M. smegmatis* and **B)** PDRPv exposed *M. smegmatis*. Measurements were taken from three independent biological samples. Data are plotted as mean ± SD.

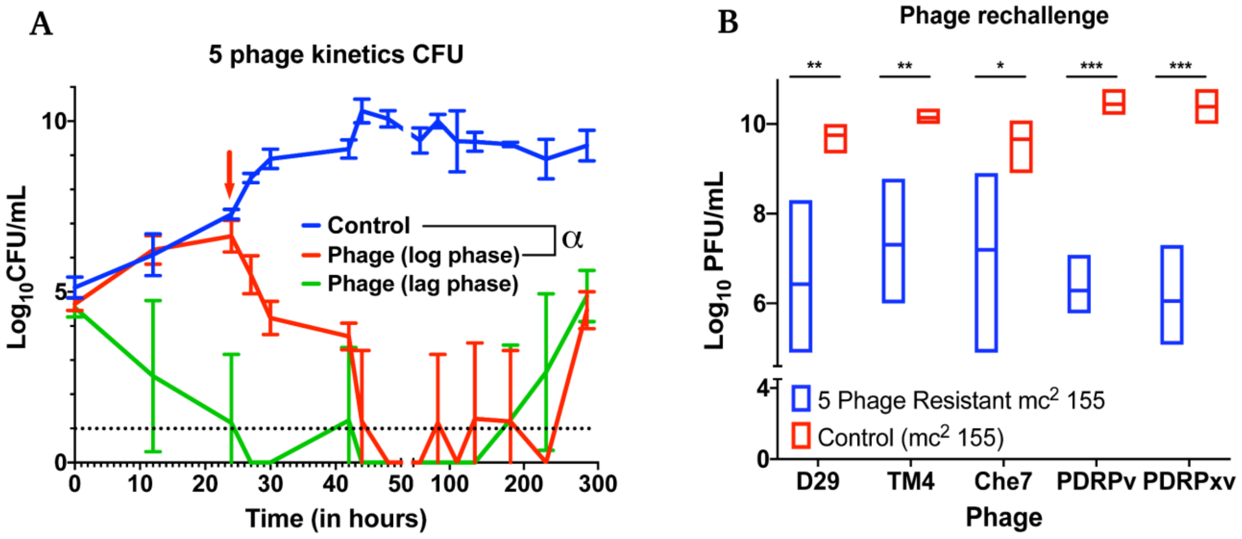

Supplementary figure S3. Phage cocktail show synergy compared to individual phage and its efficacy is MOI dependent. A) Colony Forming Unit (CFU) measurements of *M. smegmatis* over time for the 5-phage cocktail (D29, TM4, Che7, PDRPv and PDRPxv) against a non-treated control. α represents a significant difference ($p<0.05$) between control and phage (log phase) for all time points between 27

h and 285 h. Red arrow represents the time point phage solution was added to the cultures. **B)** Individual phage titers against the phage resistant growth observed in the 5-phage kinetics. Multiple t-test with Holm-Sidak method was used to determine the statistical significance (*P < .05; **P < .01; ***P < .001). The detection limit for the assay is represented in black dotted line (10 CFU). Measurements were taken from three independent biological samples. Data are plotted as mean with floating bars (min to max).

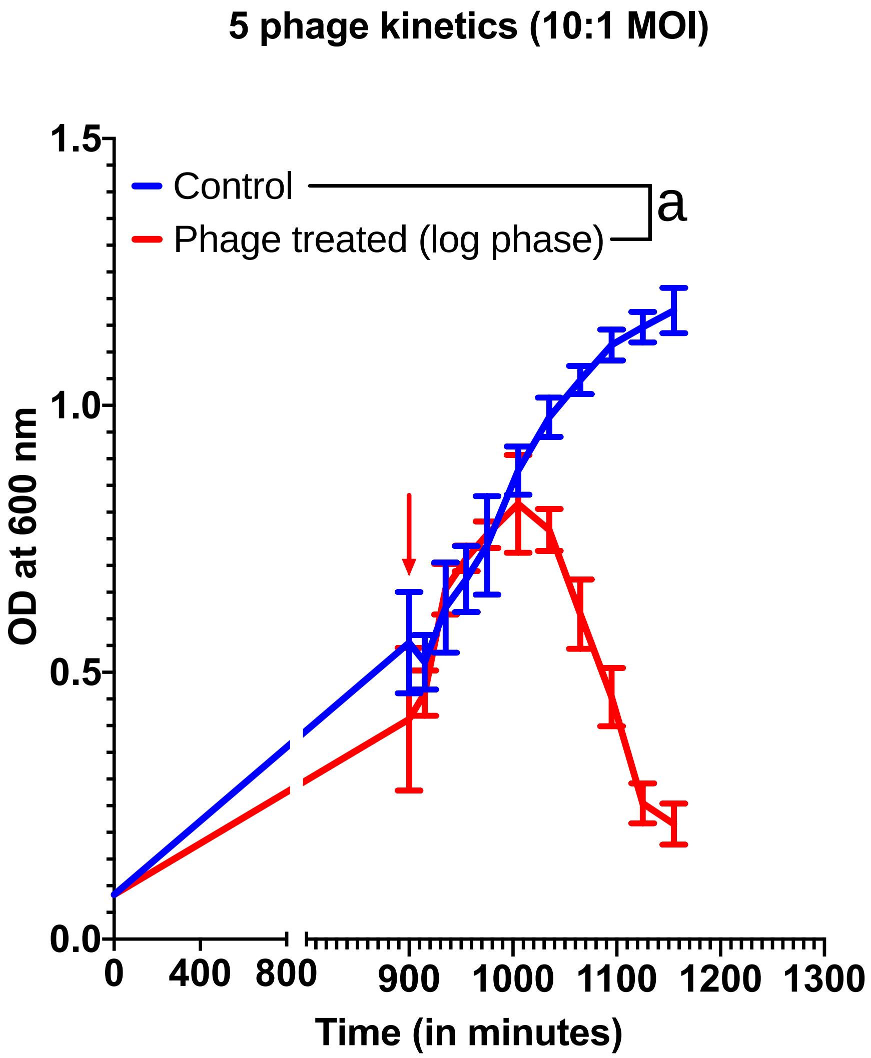

Supplementary Figure S4. Phage cocktail (D29, TM4, Che7, PDRPv and PDRPxv) kinetics at 10 MOI. Red arrow represents the time point phage solution was added to the *M. smegmatis* cultures. Multiple t-test with Holm-Sidak method was used to estimate the statistical significance ($p<0.05$). α

represents a significant difference ($p<0.05$) between control and five-phage cocktail (D29, TM4, Che7, PDRPv and PDRPxv) treated (log phase) for all time points between 1080 min and 1200 min.

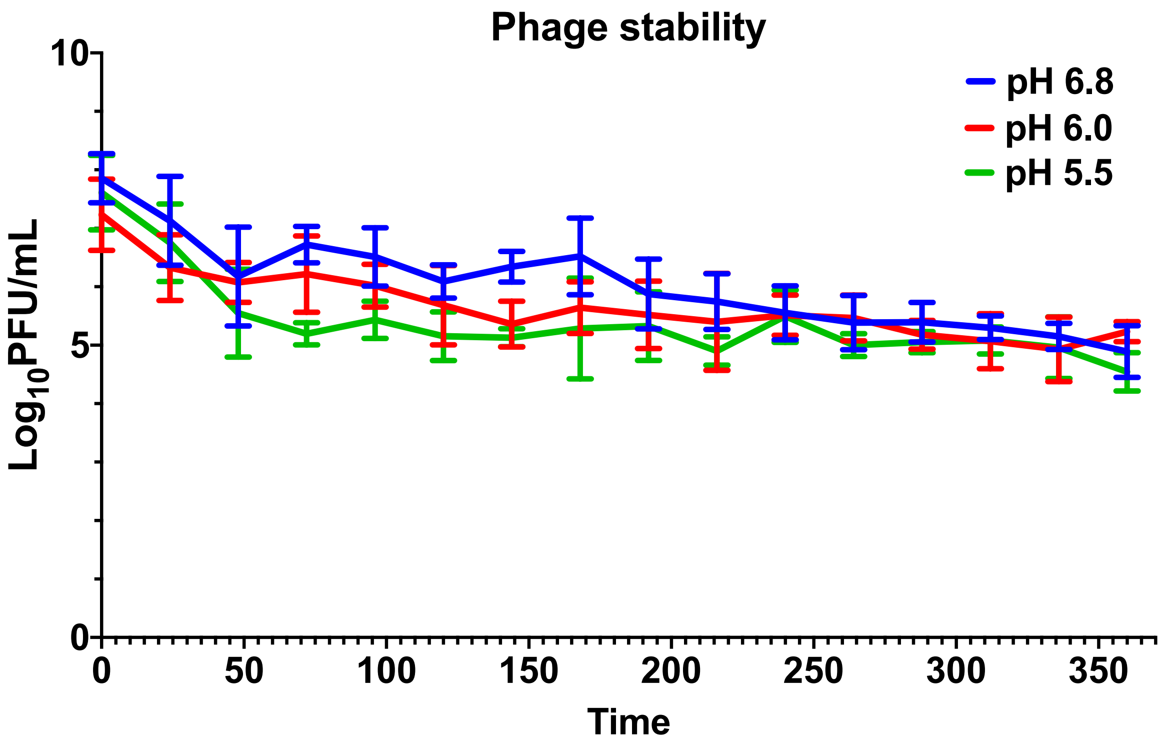

Supplementary Figure S5. Stability of phages at various pH in 7H9 medium over time.

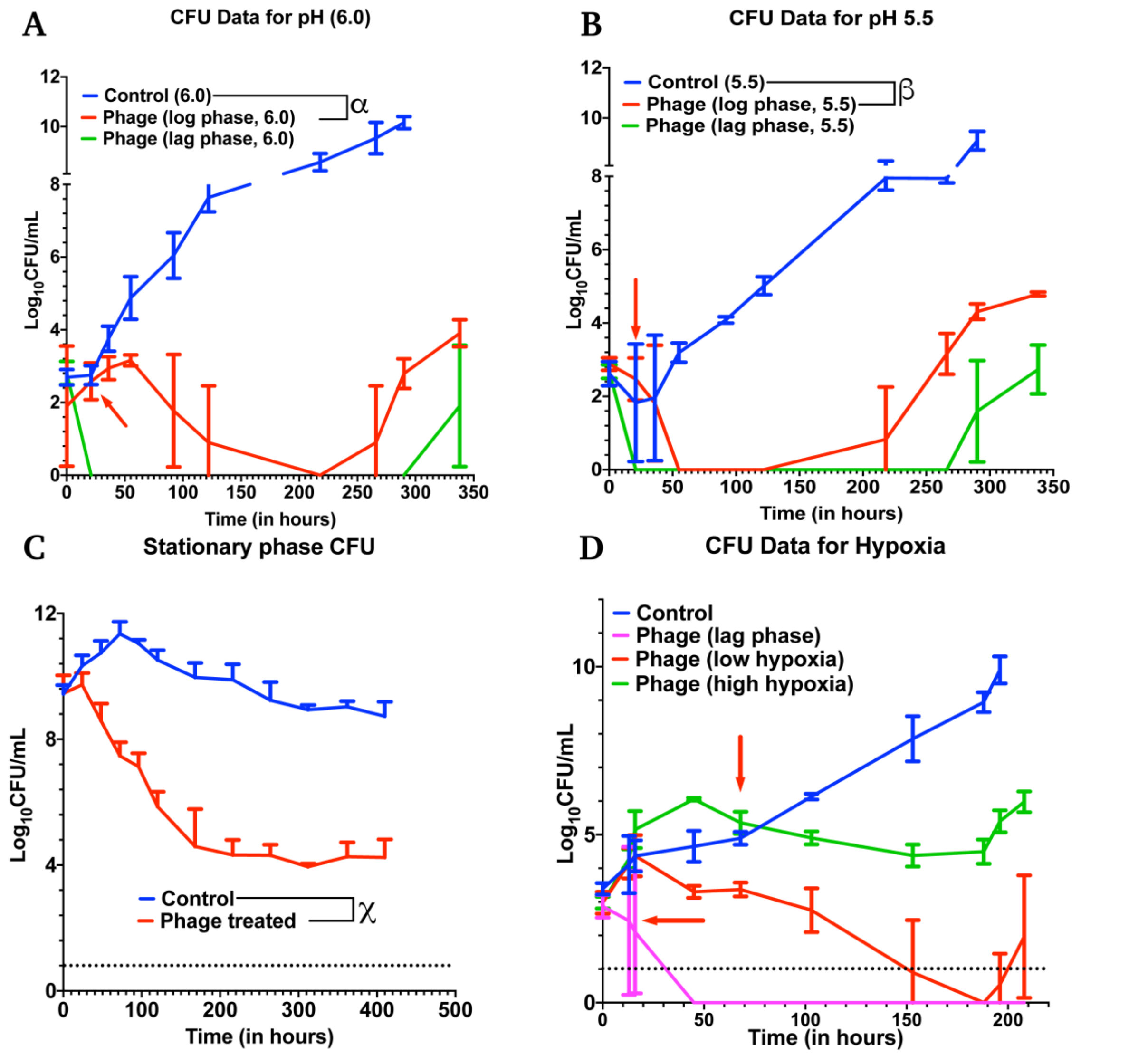

Supplementary figure S6. Mycobacteriophages are effective under various disease-mimicking conditions. Colony Forming Unit (CFU) measurements over time of *M. smegmatis* cultures treated with a 5-phage cocktail at **A)** pH condition of 6.0, **B)** pH condition of 5.5, **C)** stationary phase, and **D)** hypoxic conditions. Red arrow represents the time point phage solution was added to the cultures treated in the log phase. For **A**, **B**, and **C**, multiple t-test with Holm-Sidak method was used to estimate the statistical significance. α represents a significant difference ($p<0.05$) between control and phage treated (pH 6.0, log phase) at all time points between 55 h and 290 h. β represents a significant difference ($p<0.05$) between control and phage treated (pH 5.5, log phase) at all time points between 55 h and 290 h. χ represents a significant difference ($p<0.05$) between control and phage treated (stationary phase) at all time points between 48 h and 410 h. For **D,** 2-way ANOVA with Sidak's multiple

comparisons test was used ($p<0.05$). δ represents a significant difference between control and phage (at low hypoxia) at all time points between 45 h and 196 h. ε represents a significant difference between control and phage (at high hypoxia) at all time points between 103 h and 196 h. The detection limits for the assays are represented in black dotted line (10 CFU).

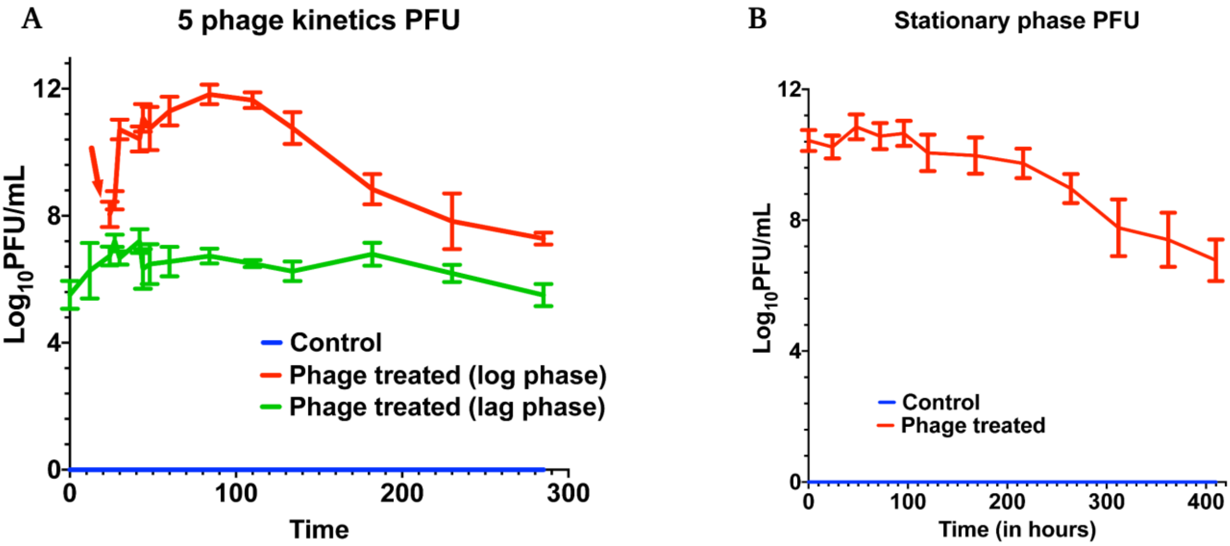

Supplementary figure S7. Phages amplify and persist throughout the duration of the experiment. Plaque Forming Unit (CFU) measurements over time of M. smegmatis **A)** lag and log phase cultures treated with 5-phage cocktail and **B)** stationary phase cultures treated with 5-phage cocktail. Red arrow represents the time point phage solution was added to the cultures treated in the log phase.

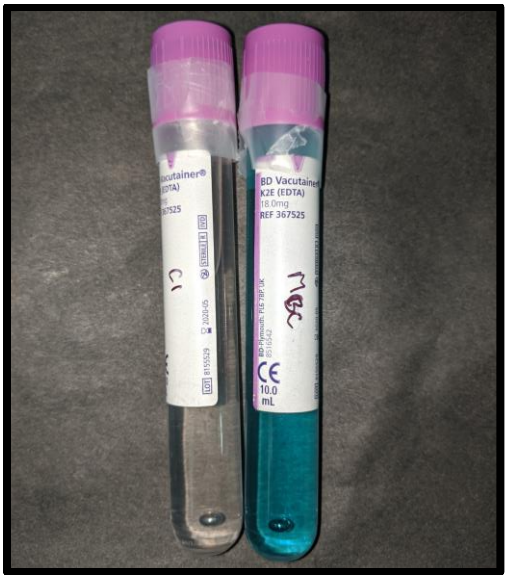

Supplementary figure S8. Methylene Blue as an indicator for hypoxia. Photograph of vaccutainers with *M. smegmatis* culture. On the left, an overnight grown *M. smegmatis* culture. Methylene Blue discolouration indicates that the culture has reached hypoxia. On the right, media after overnight incubation without bacteria where no discolouration is observed.

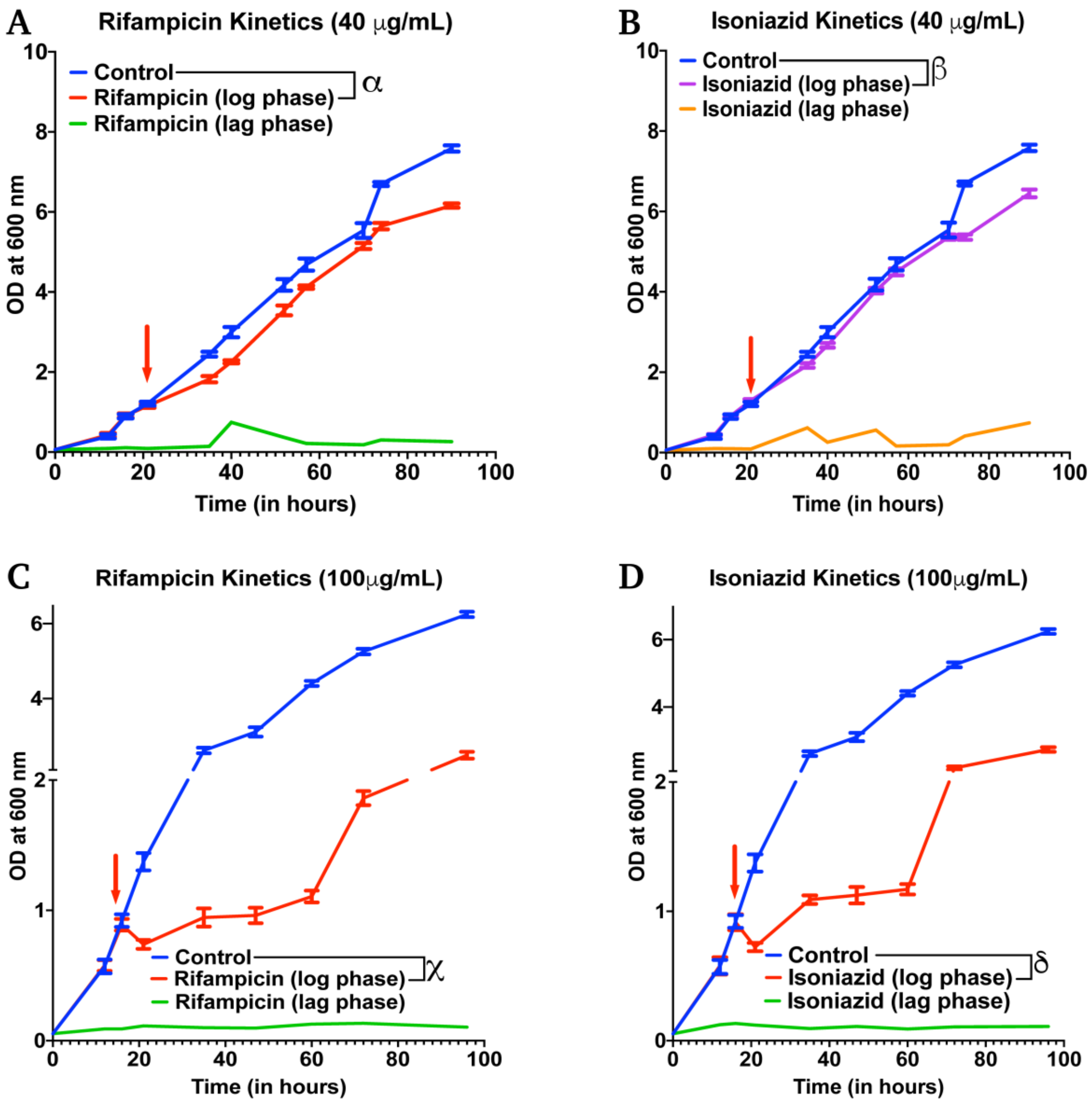

Supplementary figure S9. Growth kinetics of *M. smegmatis* cultures under antibiotic treatments. Growth kinetics of *M. smegmatis cultures* treated with **A)** rifampicin (40 μg/mL) - α represents a significant difference ($p<0.05$) between control and rifampicin treated (log phase) at all time points between 35 h and 90 h, excluding 70 h, **B)** isoniazid (40 μg/mL)- β represents a significant difference ($p<0.05$) between control and isoniazid treated (log phase) at all, **C)** rifampicin (100 μg/mL)- χ represents a significant difference ($p<0.05$) between control and rifampicin treated (log phase) for all time points between 21 h and 96 h, **D)** isoniazid (100 μg/mL)- δ represents a significant difference ($p<0.05$) between control and isoniazid treated (log phase) for all time points between 21 h and 96 h. Red arrow represents the time point antibiotic was added to the cultures. time points between 74 h and 90 h.

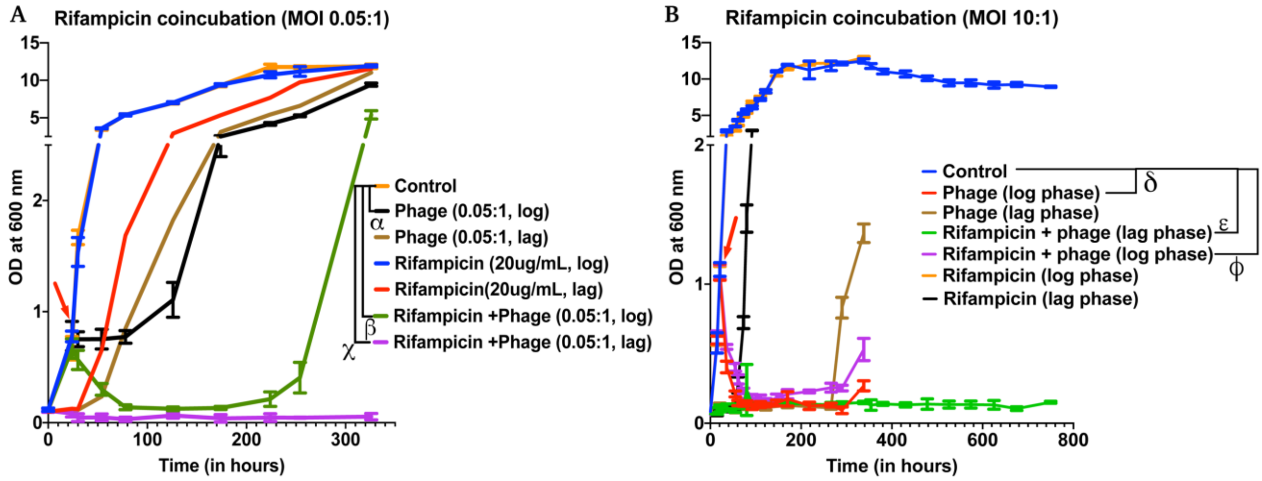

Supplementary figure S10. Rifampicin (20 µg/mL) and phage coincubation. **A)** at 5-phage cocktail MOI of 0.05- α**, and** β represent a significant difference between control and phage treated (log phase) or rifampicin + phage (log phase) for all time points between 30 h and 326 h. χ represents a significant difference between control and rifampicin + phage (lag phase) for all time points between 24 h and 326 h. **B)** at 5-phage cocktail MOI of 10- δ represents a significant difference ($p<0.05$) between control and phage cocktail treated (log phase) at all time points between 36 h and 338 h. ε represents a significant difference ($p<0.05$) between control and rifampicin + phage treated (lag phase) at all time points between 15 h and 750 h. ϕ represents a significant difference ($p<0.05$) between control and rifampicin + phage treated (log phase) for all time points between 36 h and 338 h. Red arrow represents the time point phage+ rifampicin solution was added to the cultures treated at log phase. For A, 2-way ANOVA with Sidak's multiple comparisons test was used to determine statistical significance ($p<0.05$). For B, multiple t-test with Holm-Sidak method was used to estimate the statistical significance ($p<0.05$).

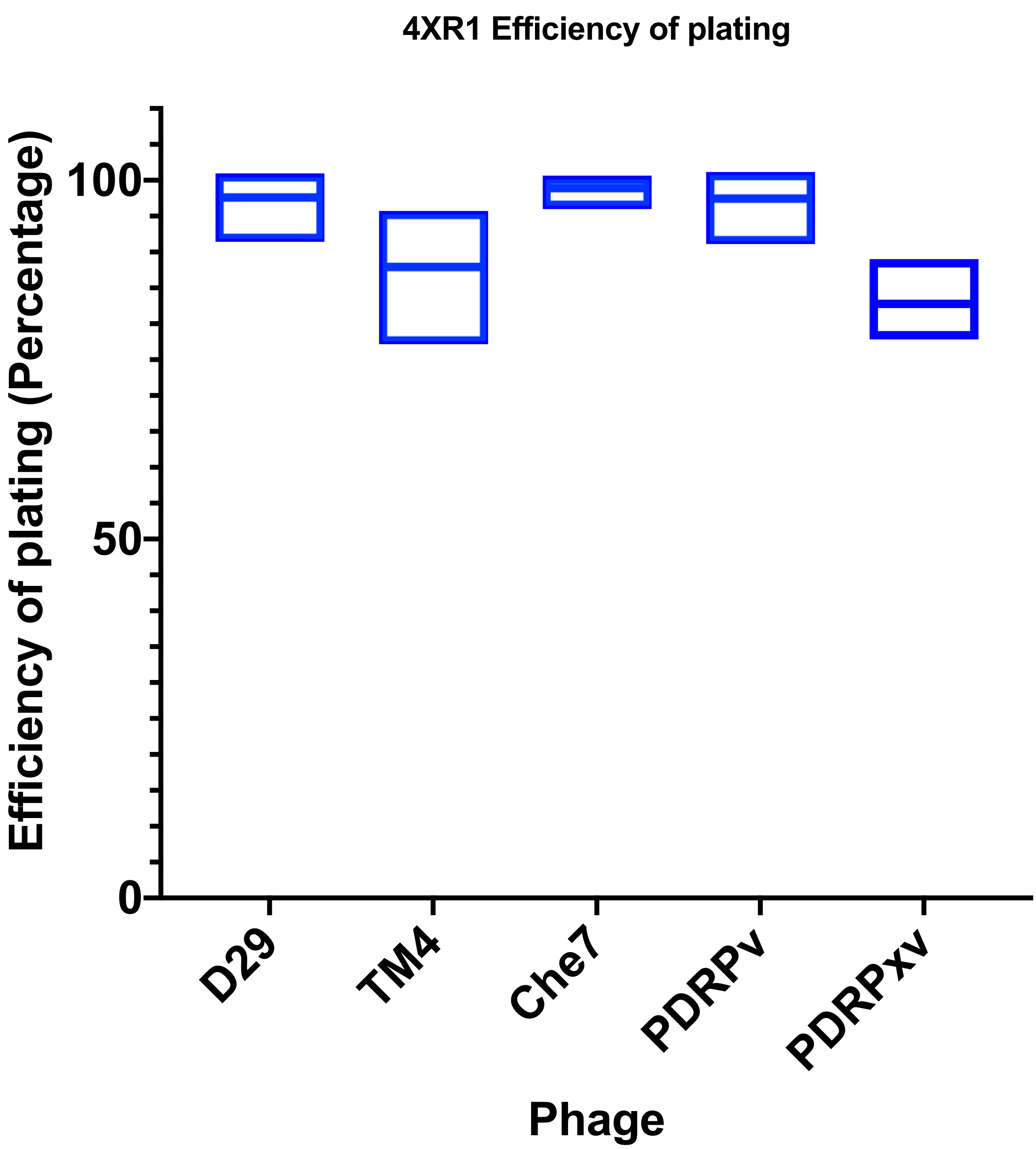

Supplementary Figure S11. Phage efficiency of plating for isoniazid resistant M. smegmatis (4XR1 strain). Experiments were carried out in triplicates (n=3). Values are normalized against phage titers against *M. smegmatis* mc[2] 155.

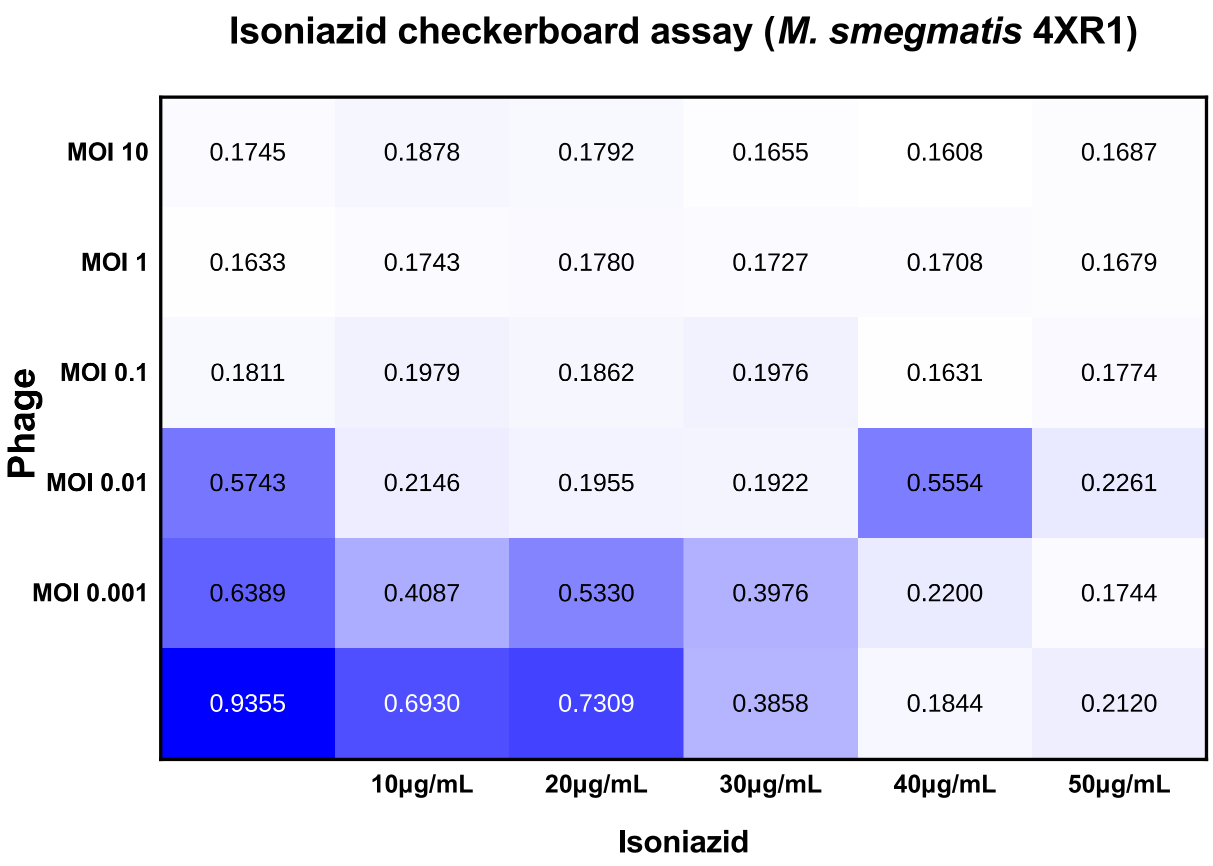

Supplementary Figure S12. Checkerboard assay of M. smegmatis 4XR1 cultures treated with isoniazid and 5-phage cocktail (D29, TM4, Che7, PDRPv and PDRPxv).
